# Corosolic acid inhibits cancer progression by decreasing the level of CDK19-mediated *O*-GlcNAcylation in liver cancer cells

**DOI:** 10.1038/s41419-021-04164-y

**Published:** 2021-09-29

**Authors:** Congcong Zhang, Yongjie Niu, Zhixian Wang, Xin Xu, Yan Li, Lifang Ma, Jiayi Wang, Yongchun Yu

**Affiliations:** 1 Institute for Thoracic Oncology, Shanghai Chest Hospital, Shanghai Jiao Tong University, 200030 Shanghai, P.R. China; 2 Shanghai Municipal Hospital of Traditional Chinese Medicine, Shanghai University of Traditional Chinese Medicine, 200071 Shanghai, P.R. China; 3 Henan University of Chinese Medicine, 450046 Zhengzhou, P.R. China; 4 Department of Oncology, Shanghai Municipal Hospital of Traditional Chinese Medicine, Shanghai University of Traditional Chinese Medicine, 200071 Shanghai, P.R. China; 5 Department of Clinical Laboratory Medicine, Shanghai Chest Hospital, Shanghai Jiao Tong University, 200030 Shanghai, China

**Keywords:** Oncogenes, Tumour angiogenesis

## Abstract

Diabetes is an important risk factor for liver cancer, but its mechanism is unknown. Corosolic acid (CA) has been proven to have both hypoglycemic and antitumor effects, so revealing the function of CA can help us understand the relationship between diabetes and liver cancer. In previous studies, we confirmed that CA can effectively inhibit the expression of YAP, an important oncoprotein in HCC cells, and the proliferation of HCC cells. In addition, we also found that *O*-GlcNAcylation plays an indispensable role in HCC tumorigenesis. However, it is not clear whether CA can inhibit the effect of *O*-GlcNAcylation on HCC cells. In this study, the antitumor ability of CA was investigated by inhibiting the *O*-GlcNAcylation level and its corresponding mechanism. The results showed that HG (high glucose) could promote the proliferation of liver cancer cells, while CA could inhibit cell growth under HG conditions and tumor growth in a xenotransplantation model. CA can inhibit the activation of the HBP pathway and reduce the expression of YAP and OGT under HG conditions. Importantly, we found that CA can reduce YAP expression and *O*-GlcNAcylation by inhibiting the activity of CDK19. Overexpression of CDK19 partially reversed the CA-induced decrease in YAP and *O*-GlcNAcylation. This is the first evidence that CA can reduce the proliferative capacity of cells with high glucose levels and further inhibit tumor growth by inactivating the CDK19/YAP/*O*-GlcNAcylation pathway, suggesting that CA is a candidate drug for the development of treatments against diabetes-associated liver cancer.

## Introduction

Liver cancer is one of the most common malignancies worldwide; because of its high incidence and mortality, its etiology and pathogenesis have been a hot topic of discussion for many years [1]. Diabetes-associated metabolic disorders have been widely recognized as one of the major risk factors for the occurrence and prognosis of liver cancer [2, 3], and the incidence and mortality of liver cancer in patients with diabetes are significantly increased [4, 5]. In diabetes, hyperglycemia induces overactivity of the hexosylamine biosynthesis pathway (HBP), leading to increased synthesis of UDP-*N*-acetyl-d-glucosamine, a substrate that *O*-linked *N*-acetylglucosamine (GlcNAc) transferase (OGT) uses to *O*-GlcNAcylate the target protein [6]. Pathologically, aberrant *O*-GlcNAcylation is closely related to the occurrence of type 2 diabetes mellitus (T2DM), and it has been shown to stimulate tumorigenesis in various cancers [7–10]. We have previously reported that high glucose (HG)-linked increases in the *O*-GlcNAcylation of target proteins plays significant roles in the development of liver cancer [11–13], HG is able to induce *O*-GlcNAcylation and expression and Yes-associated protein (YAP) can be modified by *O*-GlcNAcylation to increase its stability. The expression of YAP, the downstream effector of the Hippo signaling pathway [14], was significantly increased in liver cancer tissues and was identified as a potent oncogene in several types of cancers [15–18]. Interestingly, we also found that YAP can positively regulate glucose uptake, the synthesis of metabolites used in the HBP, and cellular *O*-GlcNAcylation, establishing a positive feedback loop [13].

**Fig. 1:**
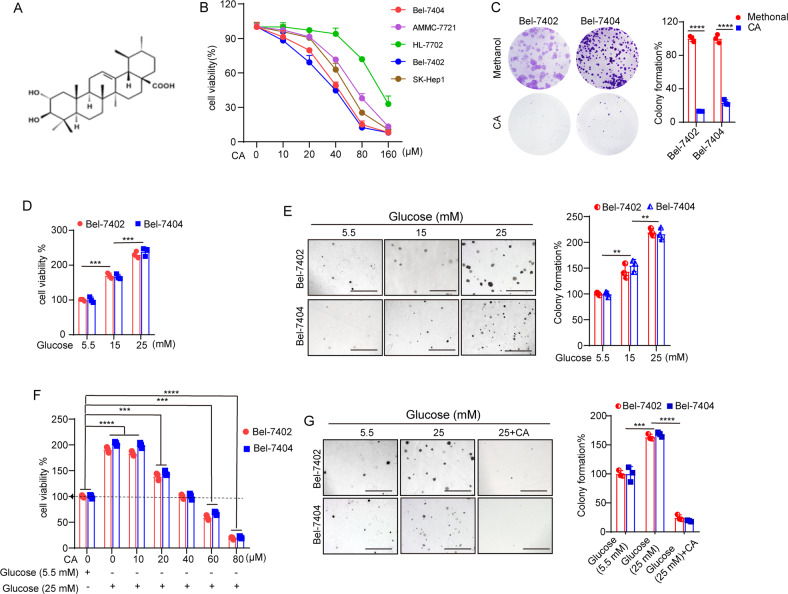
**A** The chemical structure of CA. **B** Cell viability of the indicated liver cancer cell lines (Bel-7404, SMMC-7721, SK-Hep1, and Bel-7402) and established hepatocytes (HL-7702) treated with methanol or different doses of CA was determined by CCK-8 cytotoxicity test. **C** Colony-formation assay of Bel-7402 and Bel-7404 cells treated with methanol or 40 μM CA. **D**, **E** Bel-7402 and Bel-7404 cells were cultured in medium containing the indicated concentrations of glucose before they were subjected to the CCK-8 cytotoxicity test (**D**) and soft agar colony-formation assays (**E**). Scale bars: 500 μm. **F**, **G** Cell proliferation and colony-formation capacity were measured by CCK-8 cytotoxicity tests (**F**) and soft agar colony-formation assays (**G**), respectively, in Bel-7402 and Bel-7404 cells subjected to the indicated treatments. Scale bars: 500 μm. Data are shown as the mean ± SD. **P < 0.01, ***P < 0.001, ****P < 0.0001.

Corosolic acid (CA; Fig. 1A) is a Chinese herbal monomer extracted from Valerians that has a specific chemical structure. Its molecular formula is C_30_H_48_O_4,_ and its molecular weight is 472.70 g/mol. As a prevalent pentacyclic triterpenoid, a large number of experimental studies have confirmed that CA has a significant effect against diabetes [19, 20]. For the prevention and treatment of T2DM agents, CA has entered American Food and Drug Administration (USA) phase III clinical pharmacodynamic evaluation. Recently, the antitumor effects of CA have elicited increasing attention. We have previously demonstrated that CA could promote the phosphorylation and ubiquitination of YAP, leading to a decrease in the YAP expression level [21]. In view of the relationship between YAP and *O*-GlcNAcylation in liver cancer obtained from our previous study, we hypothesized that CA has therapeutic potential in cancer therapy by inhibiting *O*-GlcNAcylation in liver cancer cells, but the exact mechanism is unknown. To date, the roles of CA in diabetes-associated liver cancer have not yet been determined. In this study, we revealed that HG positively regulates YAP and OGT expression to enhance the proliferative capacity of liver cancer cells. Furthermore, we revealed the molecular inhibitory mechanism of *O*-GlcNAcylation by CA. In addition, we found that YAP and OGT were positively associated with cyclin-dependent kinase 19 (CDK19) in liver cancer tissues and cell lines. Overall, this finding suggests that CA negatively regulates CDK19 to reduce the cellular *O*-GlcNAcylation level and suppress the proliferative capacity of liver cancer cells.

## Results

### CA inhibited the proliferation of liver cancer cells under HG conditions

To explore the activity of CA (Fig. 1A) in the proliferation of liver cancer cells, Cell Counting Kit-8 (CCK-8) assays were used to detect the viability of established hepatocytes (HL-7702) and liver cancer cells (Bel-7404, SMMC-7721, SK-Hep1, and Bel-7402) after treatment with serial concentrations of CA for 24 h. As presented in Fig. 1B, CA inhibited the proliferation of liver cancer cells in a dose-dependent manner. CA at 40 μM induced approximately 50% growth inhibition in Bel-7402 and Bel-7404 cells, so these two liver cancer cells were chosen in the subsequent experiments. Bright-field microscopy (Supplementary Fig. 1A) and clone-formation assays (Fig. 1C) showed that CA markedly inhibited Bel-7402 and Bel-7404 cell proliferation compared to that of the methanol treatment group. Flow cytometry was performed to analyze apoptosis and the cell cycle distribution of Bel-7402 and Bel-7404 cells. The results showed that 40 μM CA could promote apoptosis of liver cancer cells (Supplementary Fig. 1B), but there was no significant change in the cell cycle distribution after CA treatment (Supplementary Fig. 1C, D).

Next, we treated two liver cancer cell lines with increasing concentrations of d-glucose, and CCK-8 assays (Fig. 1D) and soft agar colony-formation assays (Fig. 1E) indicated that the cell proliferation activities were elevated by increasing the concentrations of glucose. Furthermore, the CCK-8 assay indicated that 40 μM CA could reduce the viability of liver cancer cells at 5.5 mM glucose to 25 mM glucose (Fig. 1F). This result was also verified as shown in Fig. 1G. Meanwhile, we investigated the influence of CA on the apoptosis of liver cancer cells and found that CA promoted the apoptosis of Bel-7402 and Bel-7404 cells cultured in the presence of HG (Supplementary Fig. 2A, B). In addition, immunofluorescence (IF) detection results showed that CA could increase the apoptosis of Bel-7402 and Bel-7404 cells (Supplementary Fig. 2C). However, the migration ability of Bel-7404 and Bel-7402 cells did not change significantly after CA treatment (Supplementary Fig. 3A). These results suggested that the proliferation ability of liver cancer cells was enhanced in response to HG culture, but CA could inhibit this enhanced viability.

To exclude the possibility that glucose enhances the activity of liver cancer cells as a result of osmotic stress, we performed parallel tests with l-glucose (the enantiomer of d-glucose). As expected, no change in the proliferation ability of cells cultured in l-glucose was observed (Supplementary Fig. 3B–D). Therefore, we propose that the effects of HG on the viability of liver cancer cells are independent of osmotic stress.

### HG stimulated the activation of the HBP pathway and increased *O*-GlcNAcylation and YAP expression

**Fig. 2:**
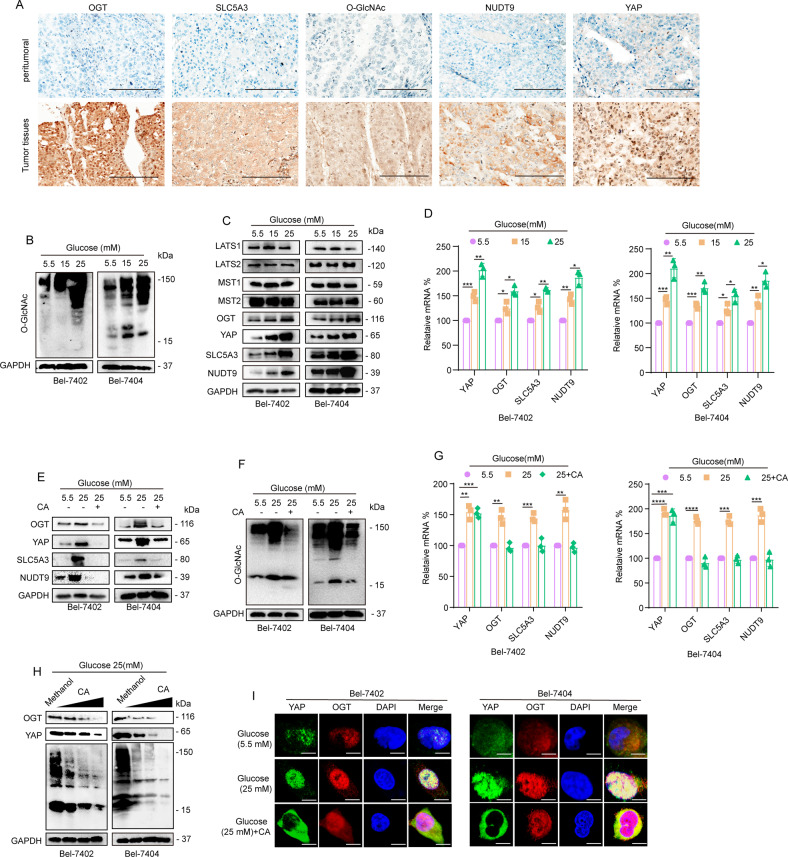
**A** Representative images of IHC staining of YAP, OGT, *O*-GlcNAc, SLC5A3, and Nudt9 in liver cancer and peritumoral tissues. Scale bar, 100 μm. Liver cancer and normal liver tissue samples were paired. **B**, **C** WB analysis of *O*-GlcNAc levels (**B**) and LATS1, LATS2, MST1, MST2, OGT, YAP, SLC5A3, and Nudt9 (**C**) expression in Bel-7402 and Bel-7404 cells cultured in media containing the indicated concentrations of glucose for 24 h. **D** YAP, OGT, Nudt9, and SLC5A3 were analyzed by qPCR in Bel-7402 or Bel-7404 cells under the indicated treatments. **E**, **F** LATS1, LATS2, MST1, MST2, *O*-GlcNAc, Nudt9, and SLC5A3 were analyzed by WB. **G** YAP, OGT, Nudt9, and SLC5A3 were analyzed by qPCR in Bel-7402 or Bel-7404 cells. **H** Representative WB images of YAP, OGT, *O*-GlcNAc, and GAPDH in Bel-7402 and Bel-7404 cells (treated with 1–40 μM CA). **I** Subcellular localization of YAP and OGT in Bel-7402 and Bel-7404 cells cultured in 5.5 mM glucose or 25 mM glucose or treated with the same amounts of methanol and CA (final concentration of 40 μM). Scale bars: 20 μm. Data are shown as the mean ± SD. *P < 0.05, **P < 0.01, ***P < 0.001, ****P < 0.0001.

Because we have previously identified the reciprocal regulation between *O*-GlcNAcylation and YAP, we mainly focused on YAP and *O*-GlcNAcylation in this study. Our previous research stated that, in an HG environment, after the level of YAP in liver cancer cells increased, global cellular *O*-GlcNAcylation could be upregulated through the regulation of Nudt9, SLC5A3, and OGT. Activation of the HBP pathway is the foundation of enhancing the *O*-GlcNAcylation level in liver cancer cells, and Nudt9 and SLC5A3 are key genes of the HBP pathway. OGT is the only known endogenous enzyme that catalyzes *O*-GlcNAcylation. Upon initial exploration of the relationship between this gene and hepatocellular carcinoma cell (HCC), we observed that YAP, OGT, SLC5A3, and Nudt9 expression in HCC tissues was upregulated relative to that in normal liver tissues (Supplementary Fig. 4A–D) through The Cancer Gene Atlas (TCGA) online visualization website. Also, we found higher levels of YAP OGT, SLC5A3, Nudt9, and *O*-GlcNAcylation in liver cancer tissues than in peritumoral tissues (Fig. 2A). Subsequently, we treated Bel-7402 and Bel-7404 cells with increasing concentrations of glucose and found that YAP, OGT, and *O*-GlcNAcylation levels (Fig. 2B–D) were elevated with increasing concentrations of glucose. Quantitative reverse transcriptase polymerase chain reaction (qRT-PCR) and western blotting (WB) results showed that the expression of SLC5A3 and Nudt9 dramatically increased in cells cultured with 25 mM glucose (Fig. 2C, D).

To exclude the possibility that HG promotes the expression of YAP by affecting the phosphorylation of LATS, we tested LATS1, LATS2, MST1, and MST2 in increasing concentrations of glucose. We found that, compared to 5.5 mM glucose, 25 mM glucose had no effect on LATS1, LATS2, MST1, or MST2 (Fig. 2C). These data suggested that the regulation of YAP by HG is not due to the effect of the Hippo pathway. Our results showed that HG promoted the expression of YAP and OGT in liver cancer cells and activated the HBP.

### CA reduced the levels of YAP and *O*-GlcNAcylation and inhibited the activation of the HBP pathway in HG

We have previously reported that CA can inhibit the expression of YAP in liver cancer cells. To investigate whether CA can inhibit the *O*-GlcNAcylation and YAP level upregulated by HG, we cultured cells with normal (5.5 mM) or high (25 mM) concentrations of glucose and treated 25 mM glucose-treated cells with CA. Because we performed relevant experiments with SMMC-7721 cells in a previous study, we also tested SMMC-7721 cells in this experiment [13]. WB results indicated that CA reduced the levels of *O*-GlcNAcylated proteins as well as the expression of YAP and OGT in Bel-7402 and Bel-7404 (Fig. 2E, F); however, in SMMC-7721 cells, CA was unable to reduce these levels as significantly in Bel-7402 and Bel-7404 cells (Supplementary Fig. 4E). In addition, CA downregulated both the mRNA and protein levels of OGT, Nudt9, and SLC5A3, which indicated that CA could inhibit the activity of the HBP pathway (Fig. 2G). We also found that CA could regulate the protein expression of YAP but had little effect on the mRNA level of YAP, indicating that CA regulates YAP expression at the protein level. These results suggest that CA can reduce the levels of YAP and OGT under HG conditions (Fig. 2G). In addition, WB assay results showed that *O*-GlcNAcylation and the expression of YAP and OGT were markedly inhibited in a dose-dependent manner by CA (Fig. 2H). These results suggested that CA could suppress the enhanced *O*-GlcNAcylation caused by HG in liver cancer cells. IF results showed that increasing concentrations of glucose can stimulate nuclear accumulation of YAP, but treatment with CA could prevent this accumulation (Fig. 2I). To test whether there is an interaction between YAP and OGT, we performed reciprocal co-immunoprecipitation (co-IP) experiments and found that OGT was dissociated from IPs pulled down by anti-YAP antibodies (Supplementary Fig. 4F) and vice versa. The results proved that YAP could interact with OGT, which was consistent with our previous research results.

### CA inhibited tumor growth in vivo

**Fig. 3:**
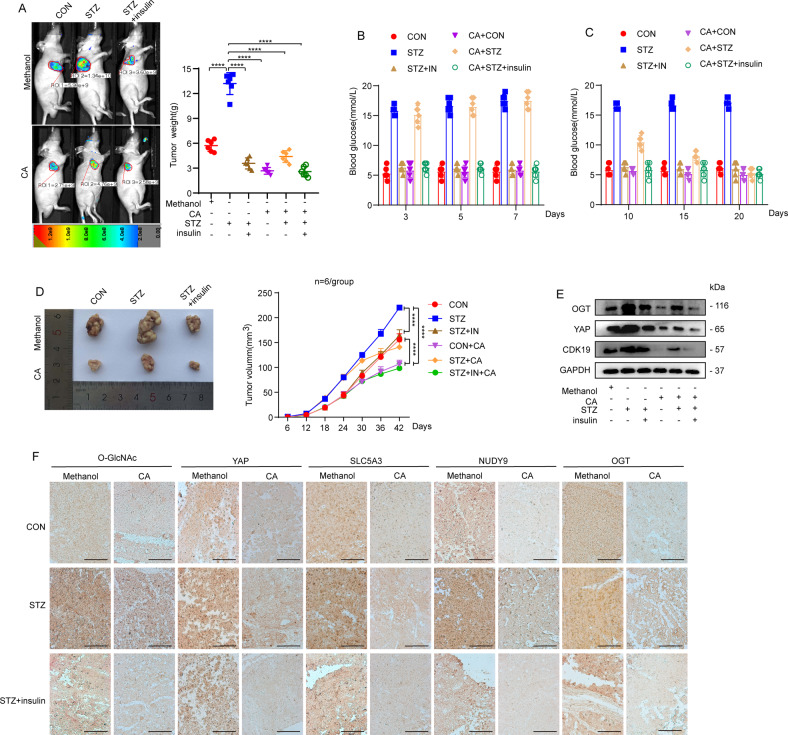
**A** Insulin-deficient HG and control mouse models were constructed by intraperitoneal injection of STZ and saline, respectively. Bel-7404 cells (5 × 10^6^ cells) expressing green fluorescent protein (GFP) were injected into 8-week-old athymic nude mice with or without STZ treatment and then injected 24 days later with methanol or CA (10 mg/kg/day). **B** Fasting blood glucose levels of mice were measured on the third, fifth, and seventh days after STZ injection. **C** Fasting blood glucose levels of mice were measured on days 10, 15, and 20 after CA injection. **D** Tumor sizes were monitored in mice for 42 days after subcutaneous injection of Bel-7404 cells. *n* = 6 per group. **E** OGT, YAP, and CDK19 were analyzed by WB of xenograft mouse tissues. **F** Representative IHC images of *O*-GlcNAc, YAP, SLC5A3, and Nudt9 staining in tissues from methanol- or CA-treated xenograft mice. Scale bars: 200 μm. Data are shown as mean ± SD. ****P < 0.0001.

To estimate the antitumor effect of CA at HG levels in vitro, we used streptozotocin (STZ) to induce insulin-deficient diabetic mice with elevated blood glucose levels (Fig. 3A). Then we generated a xenograft mouse model in Balb/c nude mice with Bel-7404 cells expressing a control green fluorescent protein (GFP) tag; 24 days later, mice were injected with diluted methanol or CA (10 mg/kg/day). We found that STZ could induce an increase in blood glucose in mice, while the blood glucose in mice treated with insulin was significantly decreased, which indicated that the insulin-deficient mouse model was successfully established. In addition, the blood glucose level in STZ-treated mice also decreased significantly after CA treatment (Fig. 3B, C). Meanwhile, STZ could induce significant in vivo growth of xenograft tumors, but treatment with CA or insulin can considerably reduce the size and weight of tumors, suggesting that HG promotes liver cancer cell proliferation but that CA can inhibit it (Fig. 3D). Further WB analysis of xenograft tissues revealed that STZ induced the expression of OGT and YAP, but CA reduced the increase in OGT and YAP induced by STZ (Fig. 3E). Immunohistochemical (IHC) staining indicated that STZ treatment induced an increase in the levels of OGT, YAP, and *O*-GlcNAcylation; moreover, the levels of the key genes of HBP (SLC5A3 and Nudt9) increased significantly, and their expression significantly decreased in the tissue upon CA treatment (Fig. 3F).

### CDK19 is involved in CA-mediated inhibition of *O*-GlcNAcylation in liver cancer cells

**Fig. 4:**
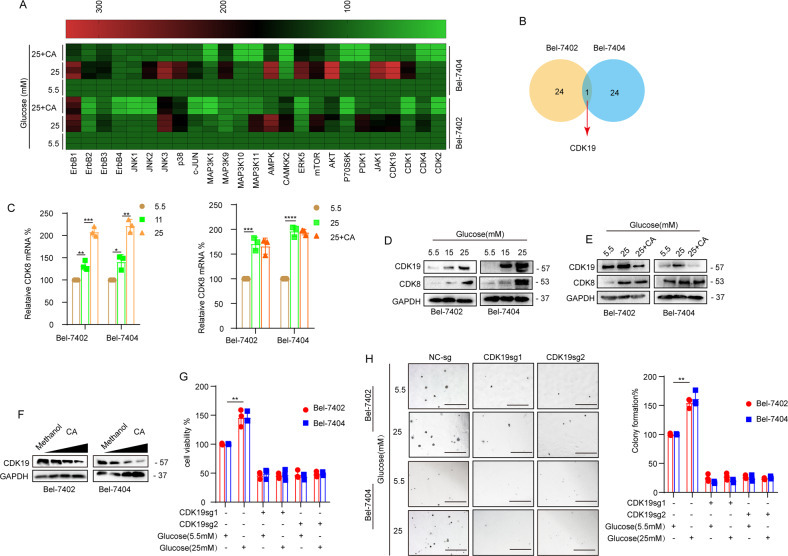
**A**, **B** RT-qPCR assay detected the mRNA expression levels of 25 different kinases in Bel-7402 and Bel-7404 cells and detailed information on one overlapping gene. **C** CDK8 was analyzed by qPCR in Bel-7402 or Bel-7404 cells subjected to the indicated treatments. **D**, **E** CDK19 and CDK8 were analyzed by WB in Bel-7402 or Bel-7404 cells subjected to the indicated treatments. **F** Representative WB images of CDK19 in Bel-7402 and Bel-7404 cells (transfected with 1–40 μM CA). **G**, **H** Cell proliferation and colony-formation capacity were measured with CCK-8 cytotoxicity tests (**G**) and soft agar colony-formation assays (**H**), respectively, in Bel-7402 and Bel-7404 cells subjected to different treatments as indicated. Scale bar, 500 μm. Data are shown as mean ± SD. *P < 0.05, **P < 0.01, ***P < 0.001, ****P < 0.0001.

Kinases are involved in the occurrence and development of cancer and affect the activities of tumor cells through different signaling pathways [22–24]. In recent years, the relationship between kinases and *O*-GlcNAcylation has received increasing attention [25]. Kinases are involved in the process of *O*-GlcNAcylation and play an important role [26]. The potentiation of synthase kinase GSK3β by *O*-GlcNAcylation will promote changes in metabolism in tumors and cells in the tumor microenvironment [27]. MEK2 associates with OGT, and total *O*-GlcNAcylation stimulates the protein stability and phosphorylation of MEK2 [28]. To investigate the effect of CA on kinase-related genes under HG conditions, we performed a qPCR assay to analyze the related genes and found a differentially expressed gene, CDK19 (Fig. 4A, B). We predicted that CDK19 expression in HCC tissues was upregulated relative to that in normal liver tissues (Supplementary Fig. 5A) through the TCGA online visualization website, and high expression of CDK19 was associated with poor prognosis (Supplementary Fig. 5B). Because CDK8 is a homologous twin kinase of CDK19, the expression of CDK8 was also detected in subsequent experiments. qPCR assays indicated that the mRNA expression level of CDK8 was elevated in cells cultured with increasing concentrations of glucose; however, its mRNA level showed no significant change after CA treatment (Fig. 4C). Next, we performed IHC to detect CDK19 in paired liver cancer tissues. Higher levels of CDK19 expression were found in liver cancer tissues than in normal tissues (Supplementary Fig. 5C). WB assay results showed that the expression of CDK19/CDK8 was elevated with increasing concentrations of glucose (Fig. 4D). Moreover, CA markedly inhibited the expression of CDK19 but had little effect on CDK8 in cells cultured in increasing concentrations of glucose (Fig. 4E, F). These results suggested that CA inhibits CDK19 expression in liver cancer cells under HG conditions. We found that STZ induced CDK19 expression in transplanted tumor tissue, while CA reduced the increase in CDK19 expression induced by STZ (Fig. 3E). In addition, we found that, compared with normal glucose concentrations, HG concentrations significantly enhanced cell proliferation and colony-formation ability; however, in CDK19-depleted liver cancer cells, HG was unable to induce an enhanced transformed phenotype (Fig. 4G, H), suggesting that CDK19 is required in HG-stimulated liver tumorigenesis.

### CDK19 promoted *O*-GlcNAcylation of liver cancer cells via regulation of OGT

**Fig. 5:**
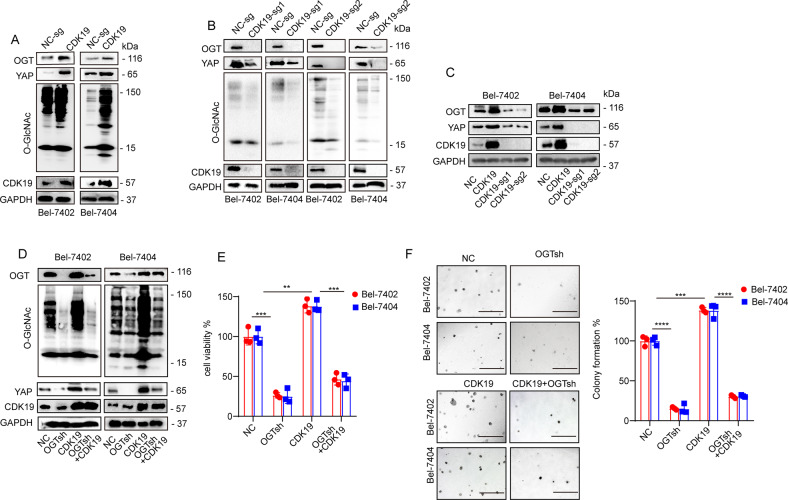
**A** Overexpression of CDK19 stimulated OGT, *O*-GlcNAc, and YAP expression, as measured by WB in Bel-7402 and Bel-7404 cells. **B**, **C** *O*-GlcNAc (**B**), OGT, YAP, and CDK19 were analyzed by WB in control cells and in Bel-7402 or Bel-7404 cells with CDK19 overexpression or knockout. **D** The protein expression of OGT, *O*-GlcNAc, YAP, and CDK19 in Bel-7402 and Bel-7404 cells was measured by WB. Cell proliferation and colony-formation capacity were measured with CCK-8 cytotoxicity tests (**E**) and soft agar colony-formation assays (**F**), respectively, in Bel-7402 and Bel-7404 cells subjected to different treatments as indicated. Scale bar, 500 μm. Data are shown as mean ± SD. **P < 0.01, ***P < 0.001, ****P < 0.0001.

To further investigate the relationship between CDK19 and *O*-GlcNAcylation, we analyzed the effect of CDK19 overexpression and knockout and found that CDK19 overexpression led to increased *O*-GlcNAcylation levels in both Bel-7402 and Bel-7404 cells, whereas CDK19 knockout had the opposite effect. Additionally, we found that OGT, the only endogenous enzyme that catalyzes *O*-GlcNAcylation, was positively regulated by CDK19 (Fig. 5A–C). Through online analysis of the GEPIA database, we found that there was a significant correlation between the expression of CDK19 and OGT in liver cancer, with a correlation coefficient of 0.44 (Supplementary Fig. 5D). Furthermore, we found that knocking down OGT led to a significant reduction in YAP and global *O*-GlcNAcylation levels, whereas overexpression of OGT upregulated the expression of YAP and global *O*-GlcNAcylation levels. However, overexpression or knockdown of OGT had no effect on CDK19 expression (Supplementary Fig. 5E). In addition, the increase in YAP and global *O*-GlcNAcylation caused by overexpression of CDK19 was reversed by simultaneous OGT knockdown (Fig. 5D). Consistent results were also verified in cell proliferation (Fig. 5E) and colony-formation assays (Fig. 5F). Collectively, these results demonstrate that CDK19 promoted cellular *O*-GlcNAcylation of liver cancer cells in an OGT-dependent manner.

### CDK19 promoted *O*-GlcNAcylation of liver cancer cells by regulating YAP

**Fig. 6:**
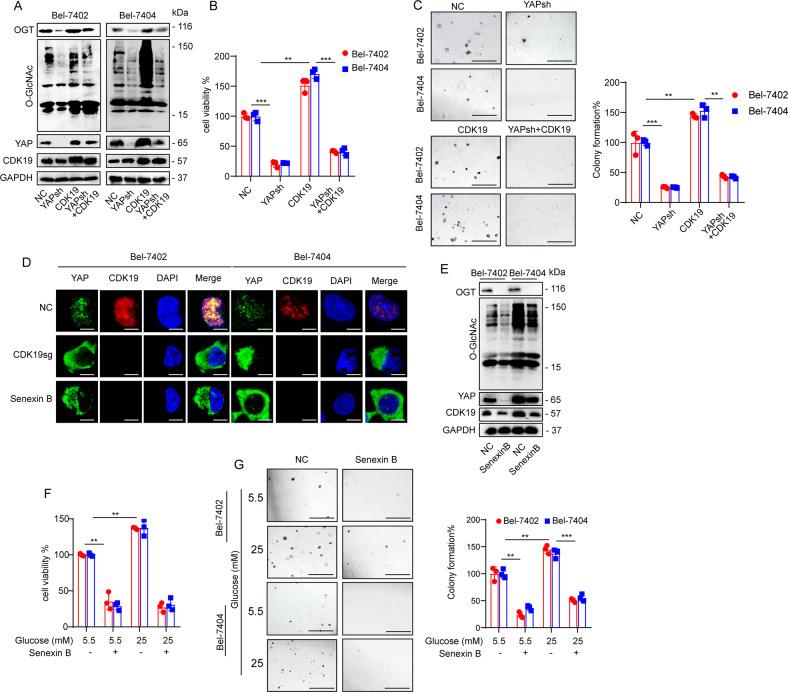
**A**–**C** The protein expression of OGT, *O*-GlcNAc, YAP, and CDK19 was measured by WB in cells subjected to the indicated treatments (**A**). Cell proliferation and colony-formation capacity were measured with CCK-8 cytotoxicity tests (**B**) and soft agar colony-formation assays (**C**), respectively, in Bel-7402 and Bel-7404 cells subjected to different treatments as indicated. Scale bar, 500 mm. **D** Subcellular localization of YAP and CDK19 in Bel-7402 and Bel-7404 cells subjected to the indicated treatments. Scale bar: 20 μm. **E** OGT, *O*-GlcNAc, YAP, and CDK19 in Bel-7402 and Bel-7404 cells subjected to different treatments as indicated were measured by WB. **F**, **G** Cell proliferation and colony-formation capacity were measured with CCK-8 cytotoxicity tests (**F**) and soft agar colony-formation assays (**G**), respectively, in Bel-7402 and Bel-7404 cells subjected to different treatments as indicated. Scale bar: 500 µm. Data are shown as mean ± SD. **P < 0.01, ***P < 0.001.

It has been shown that YAP and global cellular *O*-GlcNAcylation can establish a positive feedback loop [13], and we wondered whether CDK19 is involved in induced *O*-GlcNAcylation by regulating YAP. We found a significant correlation between CDK19 and YAP through online analysis of the GEPIA database (Supplementary Fig. 5F); therefore, we investigated whether CDK19 can regulate the expression of YAP. Indeed, overexpression of CDK19 led to a significant upregulation in YAP expression, whereas CDK19 knockout had the opposite effect (Fig. 5C). However, the contributions of YAP overexpression or knockdown to the expression of CDK19 were not significant (Supplementary Fig. 5G). In addition, YAP knockdown prevented the increase in cell *O*-GlcNAcylation caused by overexpression of CDK19. We observed that overexpression of CDK19 resulted in a significant upregulation in YAP, OGT, and cellular *O*-GlcNAcylation levels (Fig. 6A) and enhanced cell proliferation (Fig. 6B) and colony-formation capacity (Fig. 6C), whereas simultaneous knockdown of YAP reversed these effects (Fig. 6A–C). Moreover, IF experiments showed that knockout of CDK19 reduced YAP nuclear localization (Fig. 6D).

Senexin B is an inhibitor of CDK19, and via the cycloheximide (CHX) chase experiment, we found that Senexin B can reduce the stability of CDK19 (Supplementary Fig. 5H). Senexin B significantly downregulated CDK19, OGT, and YAP protein levels and *O*-GlcNAcylation levels (Fig. 6E). Additionally, Senexin B treatment resulted in a significant suppression of OGT, YAP, and *O*-GlcNAcylation levels, cell proliferation, and colony-formation capacity in both Bel-7402 and Bel-7404 cells (Fig. 6F, G). In Senexin B-treated liver cancer cells, HG was unable to induce an enhanced transformed phenotype, further illustrating that CDK19 is required in HG-stimulated liver tumorigenesis. Furthermore, we found that treatment with Senexin B resulted in a reduction in YAP nuclear localization (Fig. 6D). These results further suggest an important role for both YAP and OGT in CDK19-mediated *O*-GlcNAcylation in liver cancer cells.

### CA reduced *O*-GlcNAcylation levels by CDK19 induction

**Fig. 7:**
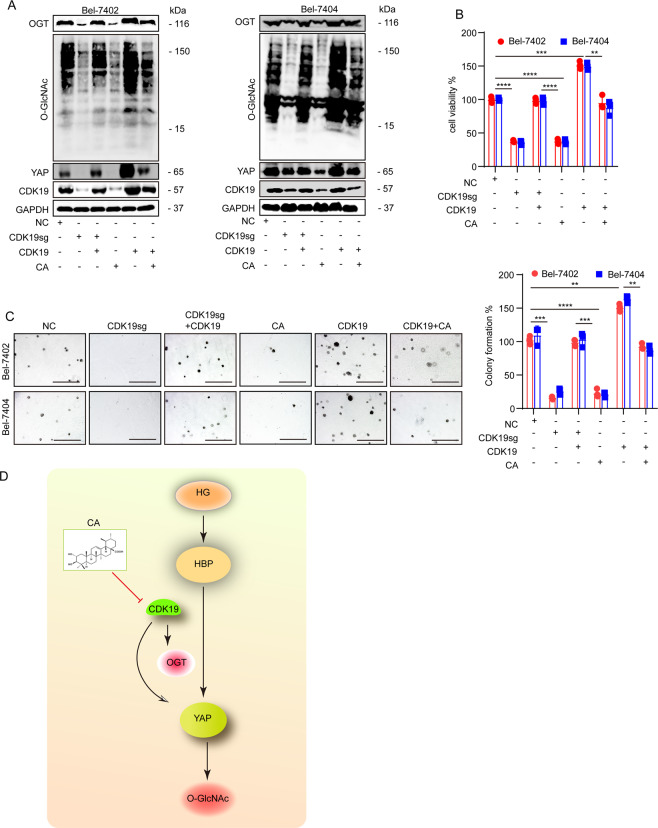
**A** The protein expression of OGT, *O*-GlcNAc, YAP, CDK19, and CDK8 was analyzed by WB in cells subjected to the indicated treatments. **B**, **C** Cell proliferation and colony-formation capacity were measured with CCK-8 cytotoxicity tests (**B**) and soft agar colony-formation assays (**C**), respectively, in Bel-7402 and Bel-7404 cells subjected to different treatments as indicated. **D** The possible mechanism of CA to inhibit diabetes-associated liver cancer. Data are shown as mean ± SD. **P < 0.01, ***P < 0.001, ****P < 0.0001.

We subsequently investigated whether CA attenuated the increase in global *O*-GlcNAcylation and inhibited the suppression of liver cancer cell proliferation through CDK19. We found that the levels of OGT, YAP, and *O*-GlcNAcylation in liver cancer cells with CDK19 knockout were increased compared to those treated with CA alone, suggesting that CA downregulates global *O*-GlcNAcylation in liver cancer cells in a CDK19-dependent manner (Supplementary Fig. 6A–C). Consistent with the previous results, we also found that CA treatment or CDK19sg treatment resulted in a significant reduction in YAP, OGT, and *O*-GlcNAcylation levels (Fig. 7A), cell proliferation (Fig. 7B), and colony-formation capacity (Fig. 7C), and overexpression of CDK19 had the opposite effect. However, impaired YAP and *O*-GlcNAcylation levels and transformative phenotypes induced by CA treatment could be partially reversed by simultaneous overexpression of CDK19. These results further suggest that CA inhibits the proliferation of liver cancer cells by inhibiting CDK19 activity and thereby decreasing the levels of YAP and *O*-GlcNAcylation.

## Discussion

CA, which is a natural flavonoid found in *Actinidia valvata* Dunn, has attracted extensive attention due to its anti-inflammatory, antioxidant, hypoglycemic, and other pharmacological effects; its antitumor effect has become a research hotspot. We have now demonstrated the antitumor effect of CA under the action of HG in vitro and in vivo, revealing the potential molecular mechanisms.

HG levels are one of the most dangerous factors for liver cancer and a major characteristic of T2DM. One analysis reported that diabetic patients have a significantly increased risk of developing liver cancer, which is associated with metastasis and poor prognosis [29] Here we found that HG can stimulate the proliferation of liver cancer tumors in both liver cancer cell cultures and xenograft models. In addition, HG can cause excessive synthesis of substrates of *O*-GlcNAcylation. As a definite oncogenic protein, YAP contributes to tumorigenesis in liver cancer. We have previously demonstrated that HG enhances the stability of YAP and intracellular levels of *O*-GlcNAcylation, leading to enhanced YAP-dependent tumorigenic phenotypes. Moreover, YAP can promote the synthesis of HBP metabolites and cellular *O*-GlcNAcylation; therefore, YAP and *O*-GlcNAcylation can promote each other to establish a positive feedback loop. Here we found that OGT, the only known endogenous enzyme that catalyzes *O*-GlcNAcylation [30, 31], was significantly upregulated in liver cancer tissues compared to adjacent normal tissues. In addition, we detected the expression of Nudt9 and SLC5A3, which are involved in HBP and are modulated by YAP [13]. We found that the expression of SLC5A3 and Nudt9 was positively associated with YAP and that their levels were elevated in liver cancer tissues. Both the mRNA and protein expression levels of the above genes in liver cancer cells are elevated with increasing concentrations of glucose. However, CA reduced the expression of Nudt9, SLC5A3, OGT, YAP, and *O*-GlcNAcylation in liver cancer cells cultured in HG. Since YAP is one of the main downstream effectors of the Hippo signaling pathway, we detected the expression of upstream genes that can control YAP in the Hippo signaling pathway in liver cancer cells [32–34]. Interestingly, the upstream genes of Hippo were almost unaffected by increasing concentrations of glucose in liver cancer cells at the mRNA and protein level. These data suggest that changes in increasing concentrations of glucose and CA treatment of YAP are independent of the Hippo pathway.

In recent years, the relationship between kinases and glycosylation has attracted much attention, and *O*-GlcNAcylation may regulate the activities of a large number of kinases. Therefore, we speculate that the mechanism by which CA regulates YAP-mediated *O*-GlcNAcylation is related to kinases. In recent years, CDK19 has been widely studied due to investigations of its important roles in transcription and oncogenesis [35, 36]. Here we found that CDK19 and *O*-GlcNAcylation were positively correlated in liver cancer tissues and cell lines. To better investigate the biological function of CDK19 in the *O*-GlcNAcylation of liver cancer cells, we knocked out or overexpressed CDK19 and found a critical role for CDK19 in *O*-GlcNAcylation levels in liver cancer cells. As a kinase, CDK19 may inhibit the phosphorylation of OGT and thus promote *O*-GlcNAcylation in liver cancer cells.

The interaction between YAP and OGT in *O*-GlcNAcylation prompted us to investigate the effect of CDK19 on YAP. Our data showed CDK19 plays an indispensable role in YAP-mediated *O*-GlcNAcylation. To further demonstrate our results, we used Senexin B to block the kinase activity of CDK19 and found that it inhibited YAP nuclear localization and cellular *O*-GlcNAcylation. Therefore, we confirmed that CDK19 participates in the regulation of *O*-GlcNAcylation in liver cancer cells, specifically through YAP and OGT. Next, we intended to determine whether the antitumor activity of CA exerted by downregulating global *O*-GlcNAcylation levels and YAP expression is dependent on CDK19. We found that CA treatment inhibited the global level of *O*-GlcNAcylation and the expression of YAP through CDK19, which eventually inhibited the transformative phenotypes of liver cancer cells. In addition, our results showed that the effects of CA on the YAP and OGT levels in SMMC-7721 cells were not very significant compared with those in Bel-7402 and Bel-7404 cells, which might be due to the heterogeneity of cell lines and the pleiotropy of CA effects. The occurrence and development of tumors is the result of a series of complex, multistep, and multifactorial interactions. Among numerous changes in the phenotypes of tumor phenotype, *O*-GlcNAcylation is only one part. There are many other factors influencing *O*-GlcNAcylation, which are reflected in similar but not identical trends in different cancer cells. CA possesses a variety of biological properties, including antidiabetic, antiobesity, antihyperlipidemia, anti-inflammatory, and anticancer effects, and can modulate a variety of cancer-related signaling pathways and processes [19, 37, 38]. Therefore, CA may inhibit cell proliferation through other previously reported targets, such as nuclear factor-κB, Wnt/β-catenin, apoptosis markers, TRIB3, and Nrf2 [39–42]. Although our findings do not rule out other possible contributions of CA mechanisms, we found that CA-mediated modulation of CDK19 is sufficient to inhibit YAP-induced cellular *O*-GlcNAcylation and proliferation under HG conditions in a cancer model.

In conclusion, our data are the first to show that high expression of CDK19 can promote the proliferation of diabetes-associated liver cancer, and the antiproliferative effect of CA in diabetes-associated liver cancer may be to inhibit the proliferation of liver cancer cells under HG conditions by decreasing the activity of CDK19 and thereby inhibiting the YAP-*O*-GlcNAcylation pathway (Fig. 7D). This evidence broadens our understanding of the antitumor mechanism of CA and suggests that CA is a potential antitumor drug for the prevention and treatment of diabetes-associated liver cancer.

## Materials and methods

### Mouse model

HG and control mouse models were constructed as described in our previous studies. In general, 36 5-week-old BALB/c male mice (SLAC; Shanghai, China) were randomly divided into groups, injected intraperitoneally with 40 mg/kg STZ (dissolved in a solution of 0.1 M citric acid, pH 4.4) or saline once a day for 4 consecutive days. STZ-treated mice were also intraperitoneally injected daily with saline or insulin (Sigma, St. Louis, MO), with a final concentration of 2 units/kg. Next, Bel-7404 (5 × 10^6^ cells) expressing GFP and subjected to different treatments were subcutaneously injected into 8-week-old athymic nude mice (SLAC) with or without STZ treatment. When the tumor mass was palpable, mice were treated with methanol or CA (10 mg/kg/day) for another 20 days via intratumoral injection. The tumor size was measured every 2 days to monitor changes in tumor growth. GFP images were obtained using a small animal in vivo imaging system, and the tumor volume was calculated using software. All mouse experiments were performed according to the institutional guidelines of Shanghai Chest Hospital.

### Tissue samples

Tumor and adjacent healthy liver tissues (Fig. 2A and Supplementary Fig. 3C) were obtained from our previous study [43]. Informed written consent was obtained from all the patients. Informed consent documents were reviewed and approved by the Ethics Committee of the Shanghai Municipal Hospital of Traditional Chinese Medicine.

### Cell culture, vectors, and drug

The liver cancer cell lines Bel-7404, HL-7702, Bel-7402, SMMC-7721, SK-Hep1, and HEK-293T were obtained from our previous study [13]. These cell lines were authenticated, and no mycoplasma contamination was detected. Cells were cultured in Dulbecco’s modified Eagle’s medium (DMEM) supplemented with 10% fetal bovine serum (FBS) and 1% penicillin–streptomycin. Cells were treated with d-glucose or l-glucose (Sigma, St Louis, MO, USA) at a final concentration from 5.5 to 55 mM, CA (Sigma, St. Louis, MO, USA) at a final concentration of 0–160 μM, Senexin B (MCE, Shanghai, China) at a final concentration of 80 nM, and CHX (Sigma) at a final concentration of 50 μg/ml.

CA solution was prepared according to the formula: mass (mg) = concentration (mM) × volume (ml) × molecular weight. In brief, 10 mg CA was dissolved in 1 ml methanol to establish a CA solution with a concentration of 20 mM that was stored at −20 °C until further use. When mice were injected with CA, the dose of CA was 10 mg/kg/day. Lentiviral-based OGT, OGT-targeted short hairpin RNA (shRNA; sh1&2), YAP, and YAP-targeted shRNA (sh1&2) were obtained from our previous studies [13]. The lentiviral-based CDK19 expression plasmid was purchased from Gene Copoeia (Heidelberg, Germany). Single-guide RNAs targeting CDK19 were constructed by PCR with the primers listed in Supplementary Table S1.

### Cell apoptosis assay

Cells were collected, washed with phosphate-buffered saline (PBS), and resuspended in buffer at a concentration of 2 × 10^6^ cells/ml. Cells were double-stained with Annexin V-FITC and propidium iodide solution (KEYGEN, Nanjing, China) and then examined by flow cytometry 10 min later.

### Cell cycle assay

The cell cycle distribution was detected with a cell cycle staining kit (MultiSciences Biotech Co., Ltd). A total of 2 × 10^5^ cells were collected, the cells were washed with PBS, and 1 ml of staining solution and 10 µl of permeabilization solution were added to resuspend the cells. The cells were stained for 30 min in the dark and detected by flow cytometry with the lowest loading rate.

### IHC and IF

For IHC, after deparaffinization and rehydration, tissue sections were treated in citrate buffer at 100 °C for 2 h to retrieve antigens. The sections were then incubated with 3% peroxide for 30 min to block endogenous peroxidase activity, blocked with 10% goat serum, and incubated at 4 °C overnight with primary antibodies. The primary antibodies used were anti-YAP (1:100, Abcam, #ab52771), anti-*O*-GlcNAc (1:200, CST, #82332 s), anti-OGT (1:100, Abcam, #ab96718), anti-SLC5A3 (1:200, ImmunoWay, #YT4344), and anti-Nudt9 (1:200, Abcam, #ab197021).

For IF, cells were fixed with 4% paraformaldehyde for 20 min, washed with PBS, incubated with blocking buffer (3% FBS, 1% HISS, and 0.1% Triton X-100) for 1 h at room temperature, and then incubated overnight at 4 °C in primary antibodies. The primary antibodies used were anti-YAP (1:100, Abcam, #ab52771), (1:100, Abcam, #ab96718), anti-CDK19 (1:100, Abcam, #ab198843), and anti-cleaved caspase substrate (1:300, CST, #8698).

### Western blotting

For WB, the proteins were resolved on sodium dodecyl sulfate–polyacrylamide gel electrophoresis gels followed by standard WB. The following primary antibodies were used: anti-MST1 (1:1000, CST, #3682), MST2 (1:1000, CST, #3952), LATS1 (1:2000, CST, #9153), LATS2 (1:1000, Abcam, #ab110780), anti-YAP, anti-OGT, anti-*O*-GlcNAc, anti-SLC5A3, anti-Nudt9, anti-CDK19, anti-CDK8 (1:500, ImmunoWay, #YT0840), and IgG (A7028 or A7016, 1:50 (IP), Beyotime).

### CCK8 and soft agar colony-formation assay

Cell viability was measured using a CCK8 (Beyotime, Shanghai, China). Cells (0.8 × 10^4^ cells in a 96-well plate) were treated with methanol or CA for 24 h. The medium was incubated with a 1:10 volume of CCK-8 solution for 1–3 h. The absorbance at 450 nm was analyzed using a microplate reader.

For the soft agar colony-formation assay, Bel-7402 and Bel-7404 cells (initially 6 × 10^3^ cells/well) were seeded into a 6-well plate with 0.3% agarose in DMEM containing 10% FBS. Colonies from 12 fields of view were counted after 2 weeks of culture.

### qRT-PCR and analysis of metabolites

Total RNA was extracted by TRIzol reagent (Invitrogen, Carlsbad, CA, USA). The primers used for qPCR are listed in Supplementary Table 2.

### Statistical analysis

Data were analyzed by GraphPad Prism 7.0. Sample size was chosen according to previous reports [13, 21]. All the data were normally distributed. Statistical analyses were performed using Student’s *t* test and two-way analysis of variance. Differences at *P* < 0.05 were considered statistically significant. Variance was similar between the groups and was statistically compared.

## Supplementary information

Supplementary information

Supplementary Table

Supplementary Figure 1

Supplementary Figure 2

Supplementary Figure 3

Supplementary Figure 4

Supplementary Figure 5

Supplementary Figure 6

## Data Availability

The data that support the findings of this study are available from the corresponding author upon reasonable request.
