# Supplementary material for: Corosolic acid inhibits cancer progression by decreasing the level of CDK19-mediated O-GlcNAcylation in liver cancer cells
Source: Cell Death Dis. 2021 Sep 29;12(10):889. doi: 10.1038/s41419-021-04164-y (PMC8481254; doi:10.1038/s41419-021-04164-y)
Supplement: Supplementary file 1 — Supplementary information [file 41419_2021_4164_MOESM1_ESM.docx]

**Supplementary information**

**Supplementary Figure 1.** (A) Brightfield images of Bel-7402 and Bel-7404 cells treated with methanol or 40 μM CA. Scale bars: 500 μm. (B) Flow cytometry was performed to analyze apoptosis in Bel-7402 and Bel-7404 cells. (C-D) Flow cytometry was performed to analyze alterations in the cell cycle distribution in Bel-7402 and Bel-7404 cells. Data are shown as the mean ± SD. ****P<0.0001.

**Supplementary Figure 2.** (A-B) Flow cytometry was performed to analyze apoptosis in Bel-7402 and Bel-7404 cells. (C) CA induced expression of cleaved caspase substrate (an apoptosis marker) as measured by immunofluorescence in Bel-7402 and Bel-7404 cells. Scale bars: 200 μm. Data are shown as the mean ± SD. ****P<0.0001.

**Supplementary Figure 3.** (A) A wound healing assay was performed to measure the migration ability of Bel-7402 and Bel-7404 cells treated with methanol or 40 μM CA. Scale bars: 500 μm. (B-D) Cell proliferation and colony formation capacity were measured by CCK-8 cytotoxicity tests (B-C) and soft agar colony formation assays (D), respectively, in Bel-7402 and Bel-7404 cells cultured in media containing the indicated concentrations of glucose. Scale bars: 500 μm. Data are shown as the mean ± SD. **P<0.01, ****P<0.0001.

**Supplementary Figure 4.** (A-D) The expression of YAP, OGT, SLC5A3 and Nudt9 in HCC and normal liver tissues was analyzed using online visualization data from UALCAN. (E). O-GlcNAc, YAP, and OGT were analyzed by WB in SMMC-7721 cells subjected to the indicated treatments. (F) Representative WB images of YAP and OGT under different processing conditions.

**Supplementary Figure 5.** (A) The expression of CDK19 in HCC and normal liver tissues was analyzed using online visualization data from UALCAN. (B) The effect of the CDK19 expression level on LIHC patient survival was analyzed using online visualization data from UALCAN. (C) Representative images of IHC staining of CDK19 in liver cancer tissues and peritumoral tissues. Scale bar, 100 μm. (D) Correlations between CDK19 expression and OGT in liver cancer were analyzed online by the GEPIA database. (E)The protein expression of OGT, YAP, and CDK19 was analyzed by WB of cells with OGT knockdown or overexpression. (F) Correlations between CDK19 expression and YAP1 in liver cancer were analyzed online by the GEPIA database. (G) The protein expression of OGT, YAP, and CDK19 was analyzed by WB of cells with YAP knockdown or overexpression. (H) CHX chase experiments of CDK19 in Bel-7402 and Bel-7404 cells treated with DMSO or Senexin B for the indicated times. The representative WB images are shown. The relative levels of CDK19 were normalized to those of GAPDH, and the value at 0 h was arbitrarily set to 100%.

**Supplementary Figure 6.** (A-C) OGT, O-GlcNAc, YAP, CDK19 and CDK8 were analyzed by WB in control cells and in Bel-7402 or Bel-7404 cells subjected to different treatments as indicated. Cell proliferation and colony formation capacity were measured with CCK-8 cytotoxicity tests (B) and soft agar colony formation assays (C), respectively, in Bel-7402 and Bel-7404 cells subjected to different treatments as indicated. Scale bar, 500 µm. Data are shown as the mean ± SD. *P<0.05, **P<0.01, ***P<0.001.
