# Supplementary material for: Corosolic acid inhibits cancer progression by decreasing the level of CDK19-mediated O-GlcNAcylation in liver cancer cells
Source: Cell Death Dis. 2021 Sep 29;12(10):889. doi: 10.1038/s41419-021-04164-y (PMC8481254; doi:10.1038/s41419-021-04164-y)
Supplement: Supplementary file 2 — Supplementary Table [file 41419_2021_4164_MOESM2_ESM.docx]

**Supplementary Table 1.**

| **Name** | **sgRNA sequence** |
| --- | --- |
| CDK19sg1: | CACCGGCAGGCAGCAGCCCCTCCAC  AAACGTGGAGGGGCTGCTGCCTGCC |
| CDK19sg2: | CACCGGGGCACCAGGCACATGGTGA  AAACTCACCATGTGCCTGGTGCCCC |

**Supplementary Table 2.**

| **Gene** | **Sequence (5′-3′)** |
| --- | --- |
| OGT F: | TCCTGATTTGTACTGTGTTCG |
| OGT R: | AAGCTACTGCAAAGTTCGGTT |
| YAP1 F: | TAGCCCTGCGTAGCCAGTTA |
| YAP1 F R: | TCATGCTTAGTCCACTGTCTGT |
| SLC5A3 F: | AGCACCGTGAGTGGATACTTC |
| SLC5A3 R: | CCCTGACCGGATGTAAATTGG |
| NUDT9 F: | AATTCTCACAATAAGGCTCGGAC |
| NUDT9 R: | CACTTTCTCATTAGGAACCTGGC |
| ErbB1 F: | AGGCACGAGTAACAAGCTCAC |
| ErbB1 R: | ATGAGGACATAACCAGCCACC |
| ErbB2 F: | TGCAGGGAAACCTGGAACTC |
| ErbB2 R: | ACAGGGGTGGTATTGTTCAGC |
| ErbB3 F: | GGTGATGGGGAACCTTGAGAT |
| ErbB3 R: | CTGTCACTTCTCGAATCCACTG |
| ErbB4 F: | GTCCAGCCCAGCGATTCTC |
| ErbB4 R: | AGAGCCACTAACACGTAGCCT |
| JNK1 F: | GGGTATGCCCAAGAGGACAGA |
| JNK1 R: | GTGTTGGAAAAGTGCGCTGG |
| JNK2 F: | GAAACTAAGCCGTCCTTTTCAGA |
| JNK2 R: | TCCAGCTCCATGTGAATAACCT |
| JNK3 F: | CAGATGGAATTAGACCATGAGCG |
| JNK3 F: | TCAATGTGCAATCAGACTTGACT |
| p38 F: | CCCGAGCGTTACCAGAACC |
| p38 F: | TCGCATGAATGATGGACTGAAAT |
| c-JUN F: | TCCAAGTGCCGAAAAAGGAAG |
| c-JUN F: | CGAGTTCTGAGCTTTCAAGGT |
| MAP3K1 F: | CATCAGGTCGCACAGTGAAAT |
| MAP3K1 F: | TCAGGGCTATATGGTGAGAAGC |
| MAP3K9 F: | GCCGTGTTCGAGTACGAGG |
| MAP3K9 F: | GGACACCTGCGAGTCCTTG |
| MAP3K10 F: | ATCGCGTCCAGGTGCTTTC |
| MAP3K10 F: | GCCCGATAGACCTTGCCAA |
| MAP3K11 F: | GCAGCCCATTGAGAGTGAC |
| MAP3K11 F: | CACTGCCCTTAGAGAAGGTGG |
| AMPK F: | TTGAAACCTGAAAATGTCCTGCT |
| AMPK F: | GGTGAGCCACAACTTGTTCTT |
| CAMKK2 F: | CGGTCGCAAGCTGTCTCTG |
| CAMKK2 F: | GCGTCCGTTCATGTCCAGG |
| LKB1 F: | ATGCTTGGAACCGGACCTG |
| LKB1 F: | TCTTGACTCATCTCTCGGAGTT |
| mTOR F: | ATGCTTGGAACCGGACCTG |
| mTOR F: | TCTTGACTCATCTCTCGGAGTT |
| AKT F: | AGCGACGTGGCTATTGTGAAG |
| AKT F: | GCCATCATTCTTGAGGAGGAAGT |
| P70S6K F: | CGGGACGGCTTTTACCCAG |
| P70S6K F: | TTTCTCACAATGTTCCATGCCA |
| PDK1 F: | CTGTGATACGGATCAGAAACCG |
| PDK1 F: | TCCACCAAACAATAAAGAGTGCT |
| JAK1 F: | CTTTGCCCTGTATGACGAGAAC |
| JAK1 F: | ACCTCATCCGGTAGTGGAGC |
| CDK19 F: | GGATTTGTTTGAGTACGAAGGGT |
| CDK19 F: | CTACAAGCCGACATGGATATTCC |
| CDK1 F: | AAACTACAGGTCAAGTGGTAGCC |
| CDK1 F: | TCCTGCATAAGCACATCCTGA |
| CDK4 F: | ATGGCTACCTCTCGATATGAGC |
| CDK4 F: | CATTGGGGACTCTCACACTCT |
| CDK2 F: | CCAGGAGTTACTTCTATGCCTGA |
| CDK2 F: | TTCATCCAGGGGAGGTACAAC |
| CDK8 F: | ACCTGTTTGAATACGAGGGCT |
| CDK8 F: | TGCCGACATAGAGATCCCAGT |
| ERK5 F: | GGTGACTTTGGTATGGCTCGT |
| ERK5 F: | CCAGAGGTCAATAGCCTGTGTA |
| CDK19 F: | GGATTTGTTTGAGTACGAAGGGT |
| CDK19 R: | CTACAAGCCGACATGGATATTCC |
| CDK8 F: | ACCTGTTTGAATACGAGGGCT |
| CDK8 R: | TGCCGACATAGAGATCCCAGT |
