# Supplementary figures and images for: Corosolic acid inhibits cancer progression by decreasing the level of CDK19-mediated O-GlcNAcylation in liver cancer cells
Source: Cell Death Dis. 2021 Sep 29;12(10):889. doi: 10.1038/s41419-021-04164-y (PMC8481254; doi:10.1038/s41419-021-04164-y)

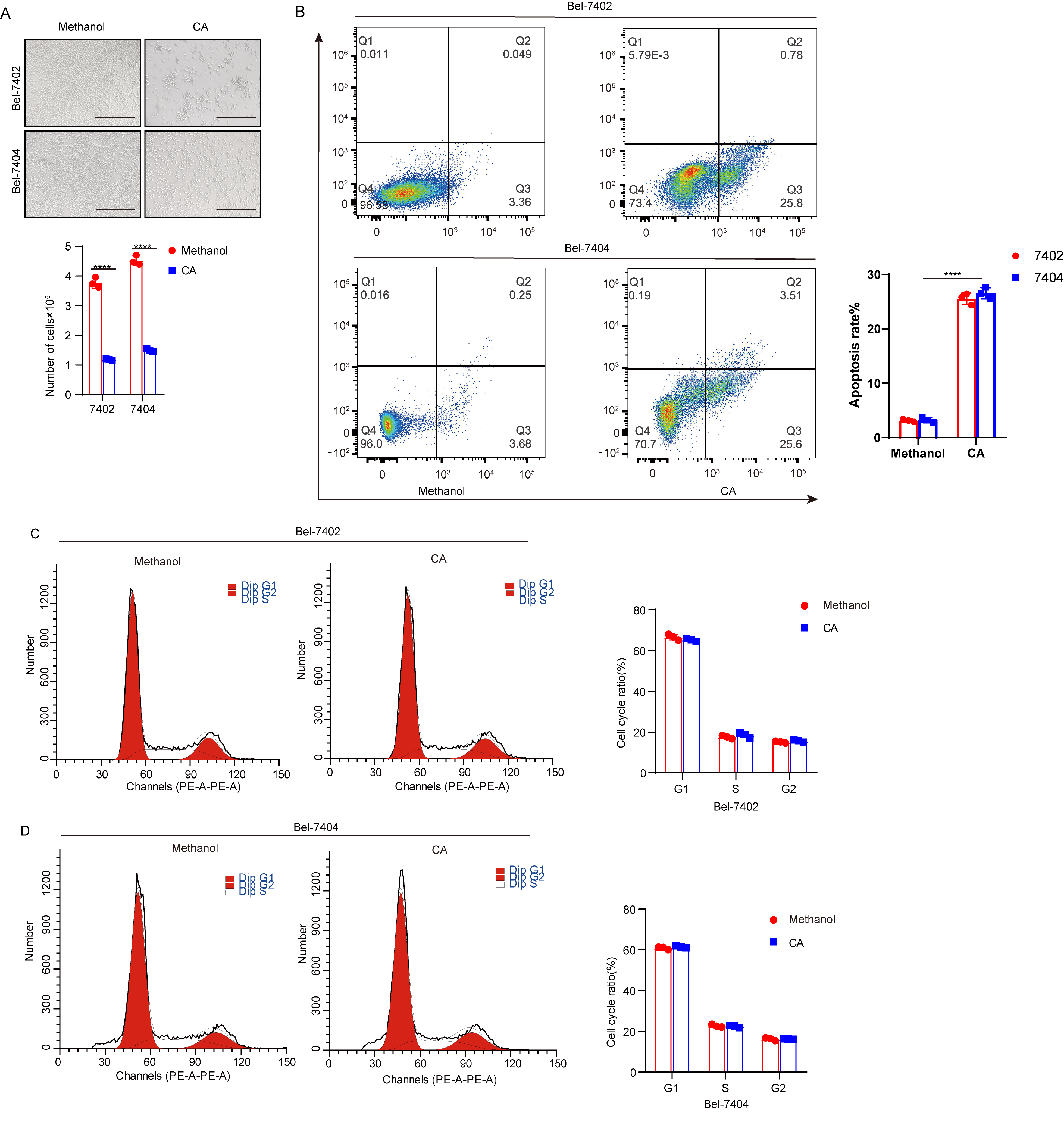

Supplement: Supplementary file 3 — Supplementary Figure 1 [file 41419_2021_4164_MOESM3_ESM.tif]

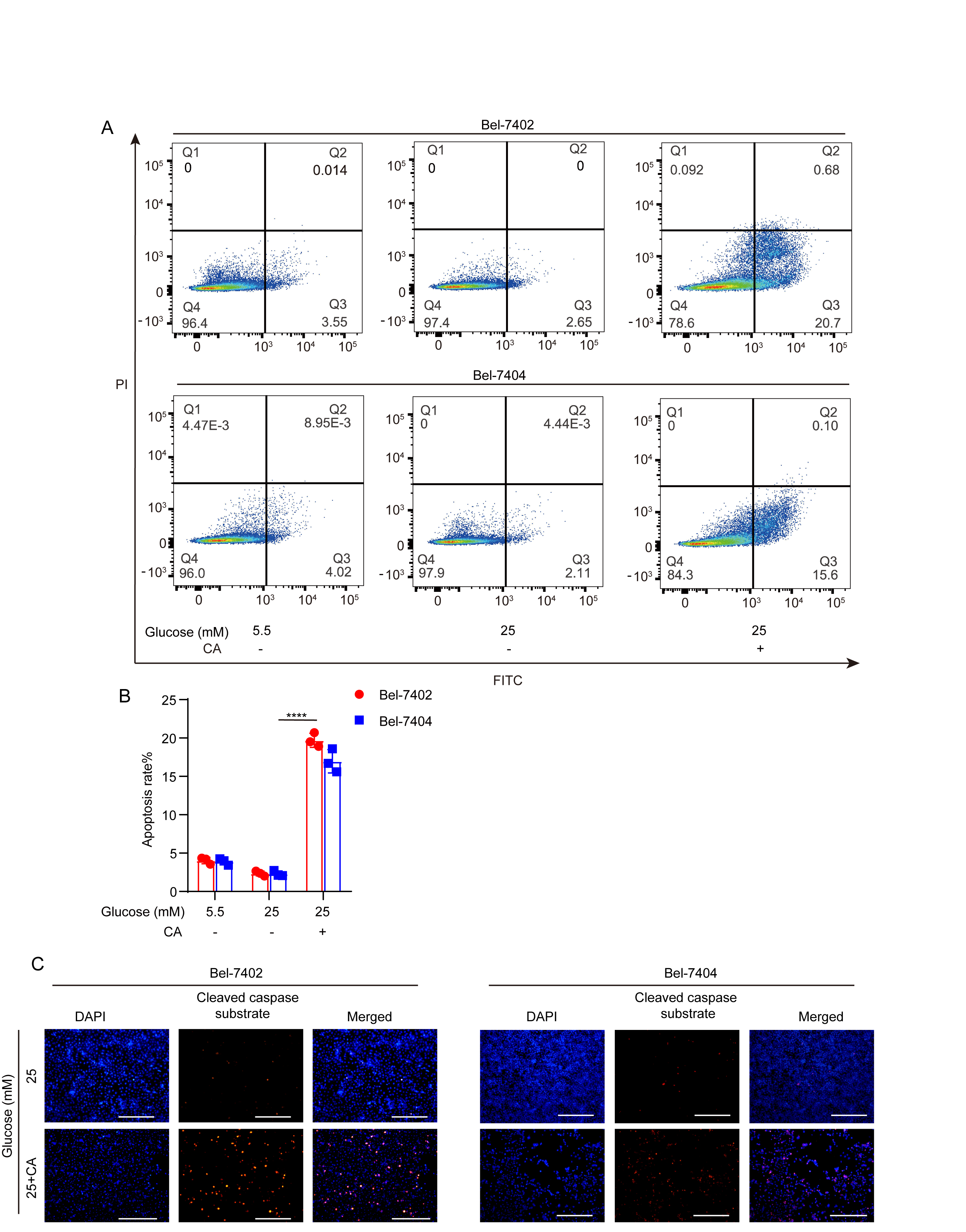

Supplement: Supplementary file 4 — Supplementary Figure 2 [file 41419_2021_4164_MOESM4_ESM.tif]

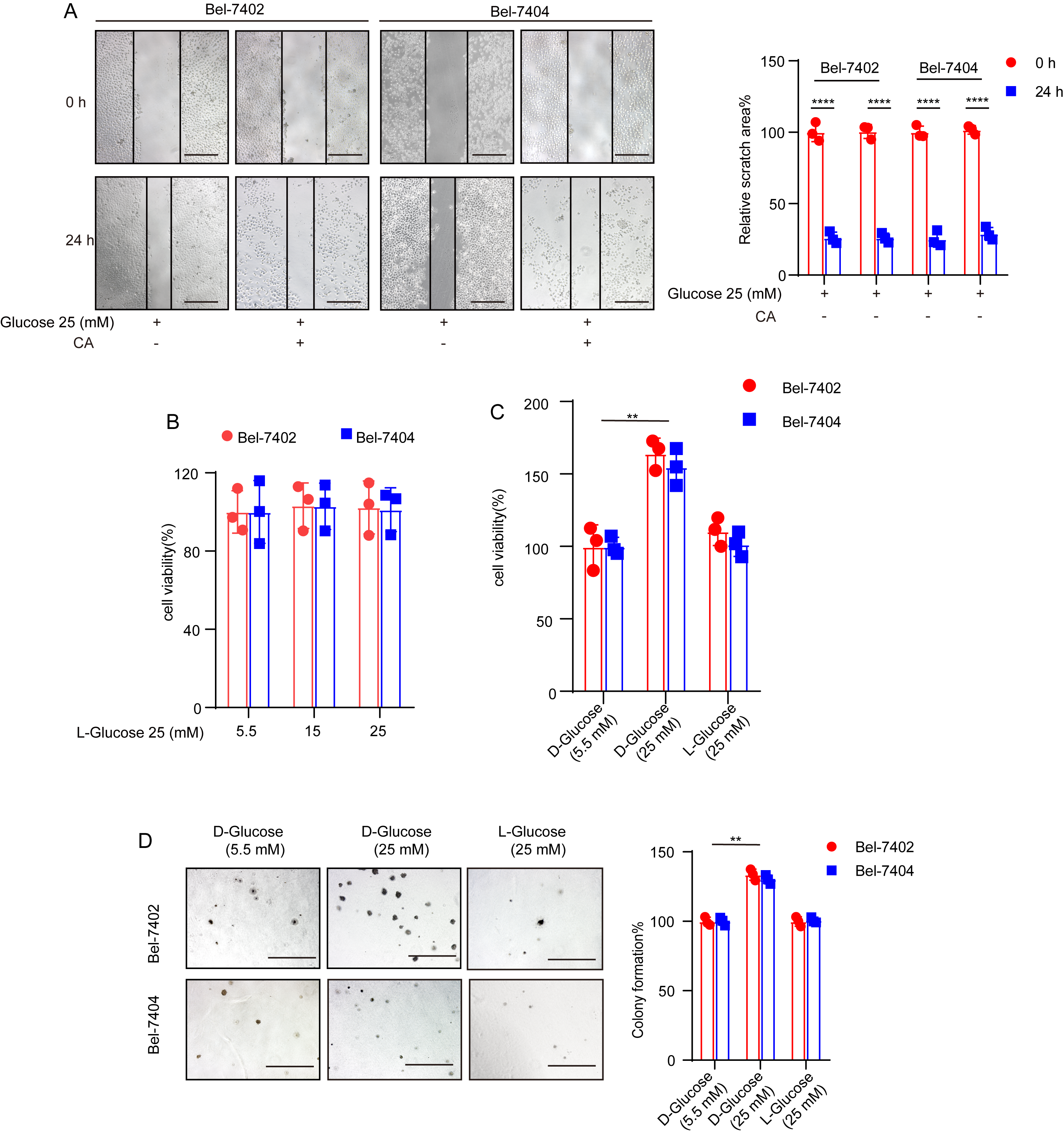

Supplement: Supplementary file 5 — Supplementary Figure 3 [file 41419_2021_4164_MOESM5_ESM.tif]

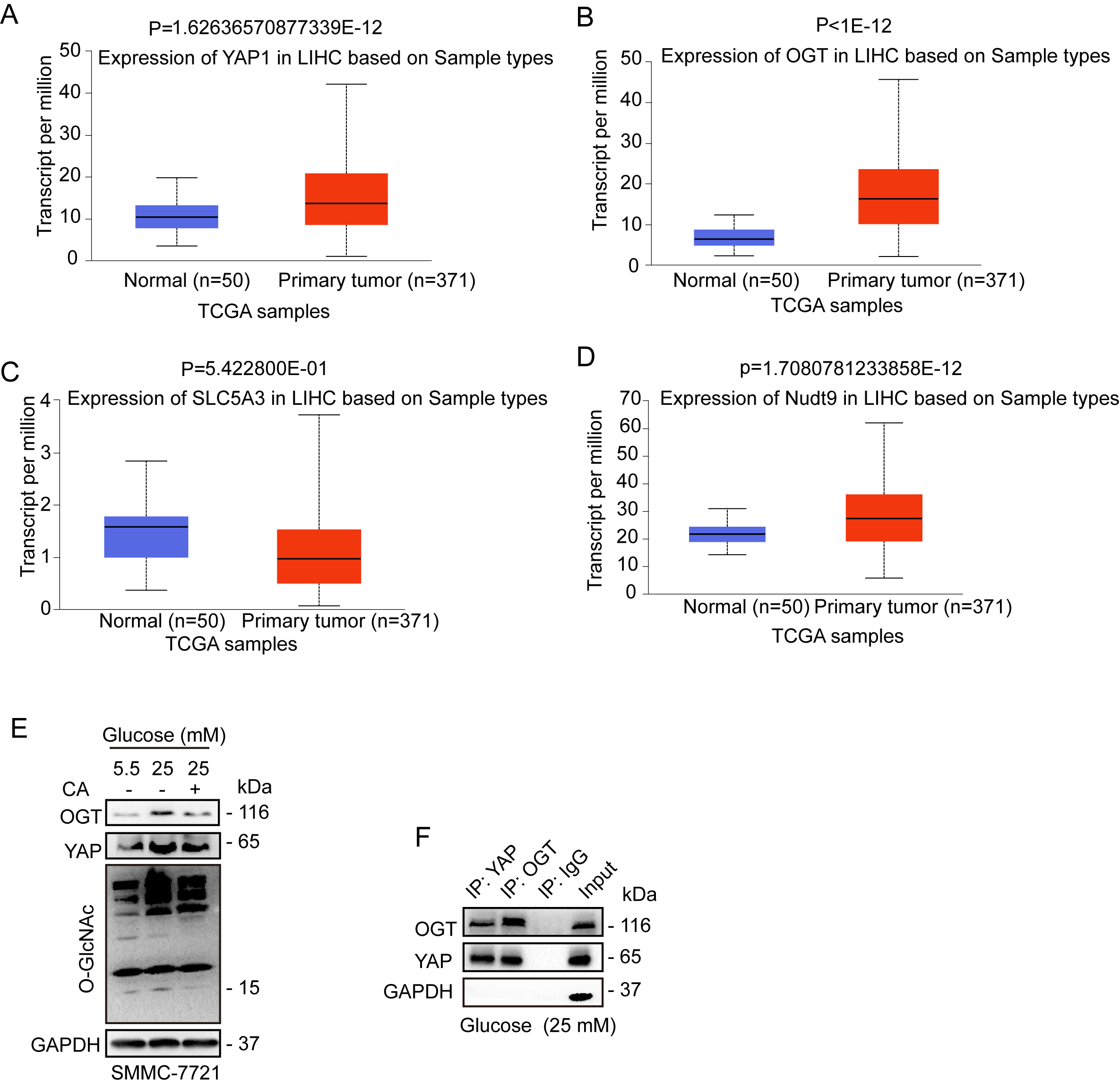

Supplement: Supplementary file 6 — Supplementary Figure 4 [file 41419_2021_4164_MOESM6_ESM.tif]

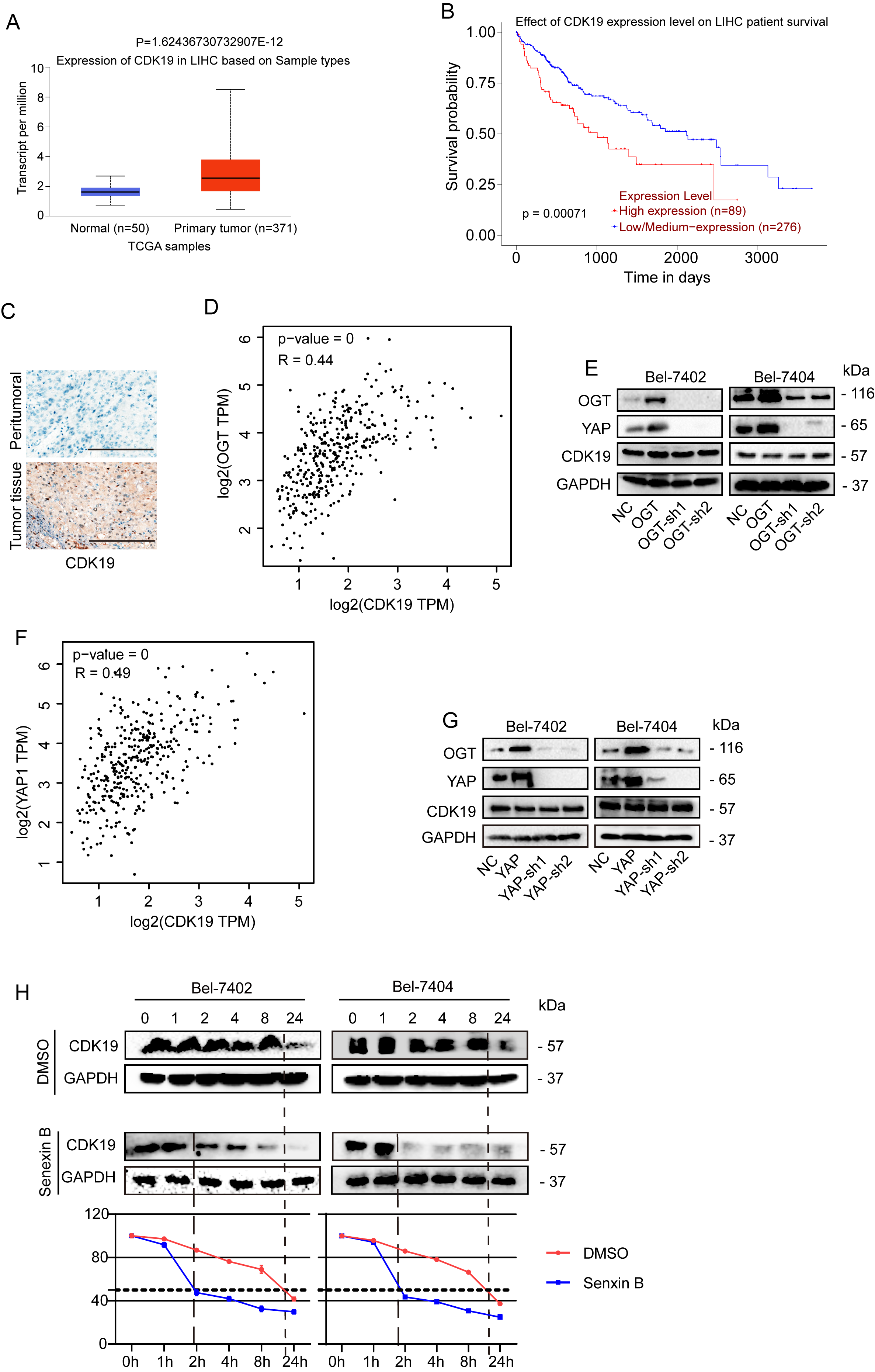

Supplement: Supplementary file 7 — Supplementary Figure 5 [file 41419_2021_4164_MOESM7_ESM.tif]

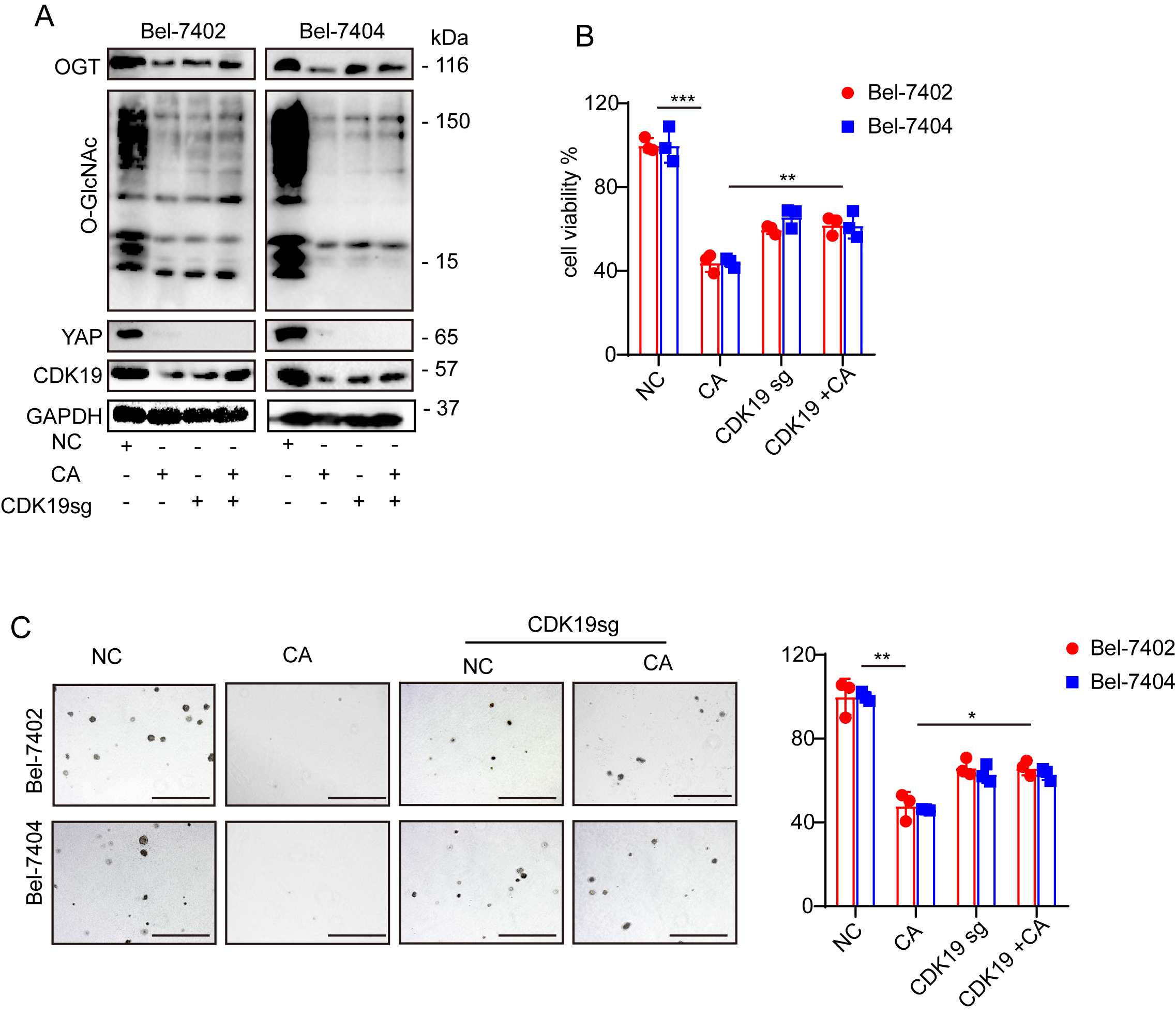

Supplement: Supplementary file 8 — Supplementary Figure 6 [file 41419_2021_4164_MOESM8_ESM.tif]
